# Evolution of structural distortion in BiFeO_3_ thin films probed by second-harmonic generation

**DOI:** 10.1038/srep38268

**Published:** 2016-12-01

**Authors:** Jie-su Wang, Kui-juan Jin, Hai-zhong Guo, Jun-xing Gu, Qian Wan, Xu He, Xiao-long Li, Xiu-lai Xu, Guo-zhen Yang

**Affiliations:** 1 Beijing National Laboratory for Condensed Matter Physics, Institute of Physics, Chinese Academy of Sciences, Beijing 100190, China; 2 University of Chinese Academy of Sciences, Beijing 100049, China; 3 Collaborative Innovation Center of Quantum Matter, Beijing 100190, China; 4 Shanghai Synchrotron Radiation Facility (SSRF), Shanghai Institute of Applied Physics, Chinese Academy of Sciences, Shanghai 201204, China

## Abstract

BiFeO_3_ thin films have drawn much attention due to its potential applications for novel magnetoelectric devices and fundamental physics in magnetoelectric coupling. However, the structural evolution of BiFeO_3_ films with thickness remains controversial. Here we use an optical second-harmonic generation technique to explore the phase-related symmetry evolution of BiFeO_3_ thin films with the variation of thickness. The crystalline structures for 60 and 180-nm-thick BiFeO_3_ thin films were characterized by high-resolution X-ray diffractometry reciprocal space mapping and the local piezoelectric response for 60-nm-thick BiFeO_3_ thin films was characterized by piezoresponse force microscopy. The present results show that the symmetry of BiFeO_3_ thin films with a thickness below 60 nm belongs to the point group *4* *mm*. We conclude that the disappearance of fourfold rotational symmetry in SHG *s*-out pattern implies for the appearance of *R-*phase. The fact that the thinner the film is, the closer to 1 the tensor element ratio *χ*_*31*_/*χ*_*15*_ tends, indicates an increase of symmetry with the decrease of thickness for BiFeO_3_ thin films.

BiFeO_3_ (BFO) has drawn considerable attention, since it has been found hitherto as the only single-phase room-temperature magnetoelectric multiferroic^1^,^2^,^3^,^4^,^5^,^6^,^7^. Meanwhile, tremendous activities have been engaged to study BFO thin films, which may meet the needs for microelectronic information storage devices^8^,^9^,^10^,^11^,^12^. BFO thin films can exhibit different structural phases, including rhombohedral-like (*R*-like) phase^13^, tetragonal-like (*T*-like) phase^14^, fully relaxed bulklike rhombohedral (*R*) phase^15^, and even fully strained tetragonal (*T*) phase^16^,^17^,^18^, depending on lattice mismatch and thermal expansion between films and different underlying substrates in the epitaxial growth progress. We speculate that the crystal structure of thin films may change from the substrate-like structure to an inclined bulklike character with the thickness of BFO thin films increasing gradually, due to the relaxation of strain, which may result in a symmetry change in BFO thin films^7^,^13^,^16^.

On the other hand, optical second-harmonic generation (SHG) is a powerful tool to explore the symmetry of noncentrosymmetric structure^19^,^20^,^21^,^22^. It works especially well when it comes to surface and interface where different media on each side break the inversion symmetry^23^, and this technique has been applied to probe many interesting and significant aspects of surface and interface, such as molecular absorption, surface electric states, and magnetic domain structure^24^,^25^,^26^. It has also been engaged in studying the structure and properties of epitaxial grown thin films, such as strain-induced ferroelectric^14^,^27^, electro-optic response^28^, surface symmetry, and interfacial enhancement^26^. So far, SHG studies of BFO thin films have been focused on single thickness of the film^14^,^29^,^30^,^31^. All this studies shine us a light for using SHG to investigate the structural evolution with thickness of ferroelectric perovskite oxide thin films. The ferroelectricity caused by the noncoincidence of positive and negative charge centers means that the centro-symmetry has been broken in the structure of material.

In this work, we reported the phase-related symmetrical evolution of epitaxial BFO thin films with various thicknesses using SHG technique. Together with X-ray diffractometry reciprocal space mapping, it is demonstrated that BFO thin films grown on the Nb-doped SrTiO_3_ (0.1 wt %, SNTO) substrates are in a *T*-like phase under a certain thickness and distort to a *R*-like one as the film thickness increases.

## Results

### Sample preparation and characterization

**Figure 1:**
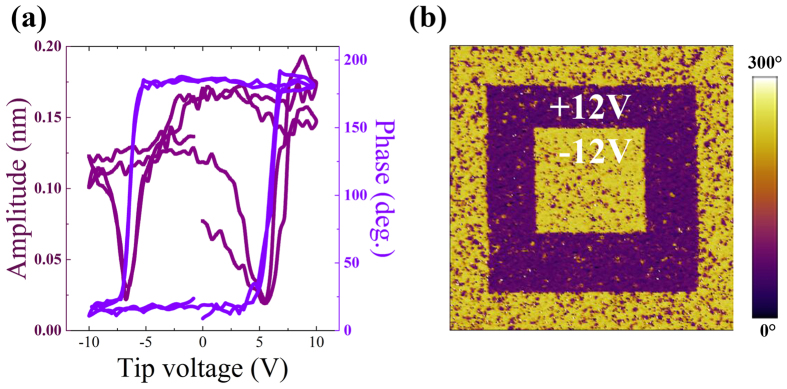
(**a**) Local PFM phase (violet line) and amplitude (purple line) hysteresis loops of 60-nm-thick BFO thin film. (**b**) Out-of-plane PFM phase image (7 μm × 7 μm) of 60-nm-thick BFO thin film after poled by DC voltage of ± 12 V.

The BFO thin films with thicknesses of 4, 12, 60, and 180 nm were epitaxially grown on SNTO substrates by a laser molecular-beam epitaxy (LMBE) system, and the detailed growth conditions are given in Methods section. The local piezoelectric response of 60-nm-thick BFO film were investigated by piezoresponse force microscopy (PFM), as shown in Fig. 1a and b. An extremely distinct polarization switching is observed for the BFO film in phase-voltage loops with almost an 180° phase change, seen in Fig. 1a. The out-of-plane PFM phase image of ferroelectric domains on 60-nm-thick BFO film surface polarized by the tip over 7 μm × 7 μm area is shown in Fig. 1b, and a sharp contrast between up and down ferroelectric domains can be clearly seen, demonstrating the ferroelectric character in 60-nm-thick BFO film.

**Figure 2:**
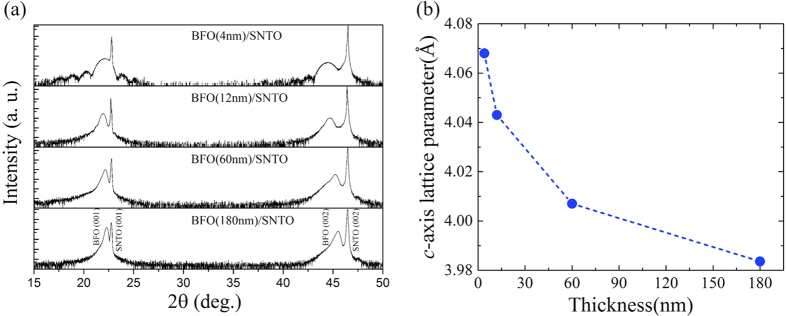
(**a**) XRD *θ–2θ* scan spectra of BFO thin films with the thicknesses of 4, 12, 60, and 180 nm grown on SNTO substrates, respectively. (**b**) *c* axis lattice parameters as a function of BFO film thickness.

The XRD *θ*–*2θ* scan curves exhibit that only (00 *l*) reflections from films and substrates were observed, as shown in Fig. 2a, without any diffraction peak from the secondary phase or the randomly oriented grains, indicating that the BFO films are epitaxially grown along *c*-axis orientation. The variation of *c* lattice parameter as a function of BFO film thickness is shown in Fig. 2b. It can be clearly seen that the out-of-plane lattice constant of BFO films decreases rapidly with increasing film thickness up to 60 nm. This can be ascribed to the relaxation of strain, which is due to the lattice mismatch between BFO films and SNTO substrates.

**Figure 3:**
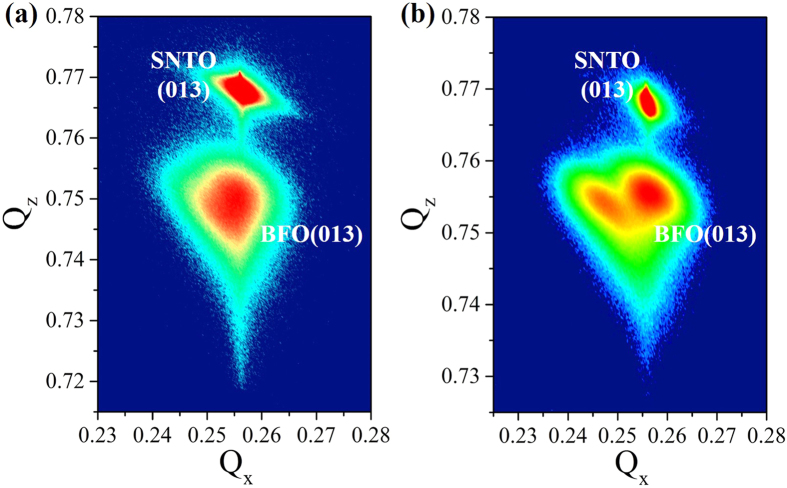
Reciprocal space mappings for (**a**) 60-nm-thick and (**b**) 180-nm-thick BFO films near SNTO (013).

The structural phases of 60 and 180-nm-thick BFO thin films were further identified by X-ray diffractometry reciprocal space mapping (RSM). It can be seen from Fig. 3 that the main BFO RSM spots almost have the same *Q*_*x*_ as that of SNTO substrate, indicating (00 l)BFO//(00 l)SNTO. The single spot at (013) diffraction for the RSM of 60-nm-thick BFO thin film, as shown in Fig. 3a, indicating a tetragonal lattice structure^16^,^32^. Similar RSM results (not shown here) were also observed both for 4-nm-thick and 12-nm-thick BFO thin films. The compressive strain coming from the mismatch between BFO films and substrates should be a main reason for BFO films to maintain a *T*-like phase with thickness less than a certain level. While in the RSM of 180-nm-thick BFO thin film, shown in Fig. 3b, one can clearly see that the diffraction spot splits into two, indicating a twinning structure. It means the film structure should be monoclinic, where four domains are supposed to exist in the thin film^32^. It is notable that a widening of the (013) diffraction peak in the RSM already appears for 60-nm-thick BFO thin film, indicating a tendency of *R*-like structure from *T*-like one. The RSM results confirm a structural phase distortion from *T*-like structure to *R*-like one for BFO thin films as the thickness increases.

### Symmetry-related SHG features

**Figure 4:**
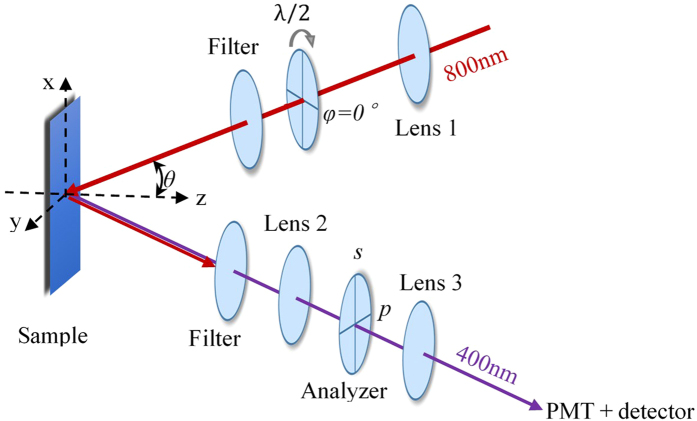
The experimental schematic for SHG reflective measurement. *θ* stands for the angle between incident light and sample normal, set as 45°. *φ* stands for the polarization angle of incident fundamental light, which is regulated by a half-wave (*λ*/2) plate assembled on a stepping motor controlled by a computer. Lens 1 and lens 3 are focusing lenses, while lens 2 is for collimating. The coordinate system is established as shown which is not sample dependent.

To further figure out the symmetry variation of BFO thin films, we carried out a systematic SHG study with a typical reflected SHG setup shown in Fig. 4. Based on different polarization combinations of incident and reflected light, two configurations were used to conduct the SHG measurement. *P*-out is denoted for the analyzer polarization parallel to the plane of incidence and incident light polarization being rotated, and *s*-out as the analyzer polarization perpendicular to the plane of incidence and incident light polarization being rotated.

**Figure 5:**
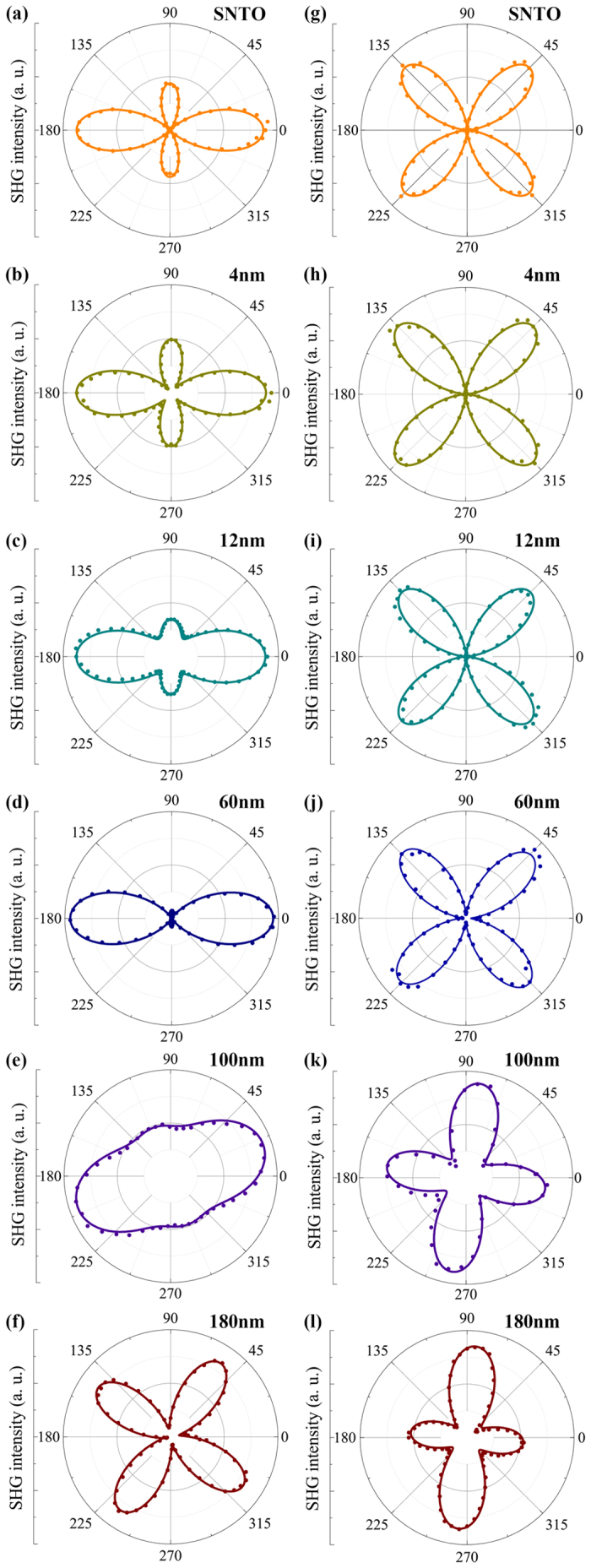
SHG polar plots. (**a**–**f**) Polar plots of measured *p*-out SHG counts depending on incident light polarization angle for (**a**) SNTO substrate and BFO films with various thicknesses of (**b**) 4, (**c**) 12, (**d**) 60, (**e**) 100 and (**f**) 180 nm, respectively. (**g–l**) Polar plots of measured *s*-out SHG counts depending on incident light polarization angle for (**g**) SNTO substrate and BFO films with various thicknesses of (**h**) 4, (**i**) 12, (**j**) 60, (**k**) 100 and (**l**) 180 nm, respectively. The colored circles show the experimental data, while simulation results are plotted with solid lines.

The SHG experimental results obtained under *p*-out and *s*-out configurations are shown in Fig. 5. For 4, 12, and 60-nm-thick BFO thin films, as shown in Fig. 5b–d, the SHG *p*-out features are characterized by a twofold rotational symmetry with two major peaks and two minor ones. The polarization angles where SHG signal peaks appear are the same, with major peaks appearing at ~0° and ~180°, and minor peaks at ~90° and ~270°, respectively. These four peaks are 90-degree symmetrical. However, the SHG intensities of the smaller bivalve peaks decrease with the increase of film thickness, and the bivalve sections at 90° and 270° are both negligibly small for 60-nm-thick BFO thin film. The peaks vanishing with the increase of film thickness suggest that some change in the allowed second-order susceptibility tensor components occurs.

The *p*-out SHG pattern of the thickest 180-nm-thick BFO film is quite different from three thinner BFO films, seen in Fig. 5f. The intensity of every SHG peak of the four-valve structures is almost equal to each other, and the peaks are symmetrically distributed at ~55°, ~145°, ~235°, and ~325°, respectively. The complete difference for 180-nm-thick BFO film from other three thinner BFO films can also be seen in SHG *s*-out features as shown in Fig. 5h–j and l. For thinner BFO films, the measured SHG *s*-out curves have fourfold rotational symmetry with the peak positions at ~45°, ~135°, ~225°, and ~315°. However, these four equal valves are totally missing for the thickest BFO thin film, as shown in Fig. 5l. Moreover, the peaks of two patterns of 180-nm-thick BFO film are not at standard angles depicted above any more, which we speculate is caused by the net polarization of sample deviating from the incident light polarization.

Comparing with the others, the features of *p*-out and *s*-out pattern of 100-nm-thick BFO film is between the thickest one and three thinner ones, as shown in Fig. 5e and k. The major peaks of its *p*-out pattern start to deviate from 0° and the four-valve structure of its *s*-out begins to rotate. Meanwhile, the relative intensity of these peaks on each pattern changes dramatically. Actually, the fourfold rotational symmetry for four-valve structure of *s*-out pattern is already broken. It should also be noticed that the minimum SHG signal is not zero any more, which could be resulted from an overlapping of SHG signals generated from different domains in *R*-like phases with different orientations in the light spot area. Since another possibility from an overlapping of those from *R*-like domains and from *T*-like domains cannot be excluded, further experiments to clarify this issue is highly expected.

### Theoretical simulation of the phase-transition-related symmetry evolution

Based on the SHG model (see Methods), we theoretically simulated the experimental results. The expression group (2), reckoned from the point group *4* *mm*, was used to stimulate the corresponding SHG features of three thinner BFO films and the results have a good agreement to the experimental data, as shown in Fig. 5b–d and Fig. 5h–j. For 180-nm-thick BFO film, this expression group is invalid to stimulate the experimental result.

As thickness increases, the strain in BFO film gradually releases, giving rise to a symmetry transition from *T*-like phase to *R*-like one in BFO thin films^16^. The structure of *R*-like BFO belongs to the point group *m*. For the 180-nm-thick BFO film, theoretical results were conducted using the expression group (3), which was reckoned from the point group *m*, denoted by solid curves in Fig. 5f and l. These solid curves have a good agreement with the corresponding experimental data, verifying that 180-nm-thick BFO films are in the *R*-like phase. For the 100-nm-thick BFO film, theoretical simulations fit the SHG results better if the point group *m* is taken.

The ratio of optical nonlinear tensor *χ*_*31*_/*χ*_*15*_ in *4* *mm* symmetry can be calculated by extracting SHG intensity at *φ* = 90° in *p*-out feature and *φ* = 45° in *s*-out feature. Details of the calculations are described in Methods. We obtained the evolution of *χ*_*31*_*/χ*_*15*_ as the increase of thickness of BFO films, as shown in Table 1. Compared with previous work for 25-nm-thick BFO film on the (110) YAlO_3_ substrate measured by 1550 nm incident light^33^, the ratio values are relatively large. We think that the difference of substrates choosing should be a main reason, and the wavelength might also affect for the large ratio. For 180-nm-thick BFO film shown in Table 1, the nonlinear coefficient ratio *χ*_*31*_/*χ*_*11*_ was acquired by extracting SHG intensity at *φ* = 90° both for its *p*-out and *s*-out features.

## Discussion

It is well known that the phase of BFO bulk is rhombohedral, and the crystal structure of ultrathin films should follow the substrates, namely *4* *mm* symmetry for SNTO surface. Therefore it can be easily understood that the structure of BFO thin film would transfer from that consisting with substrate (*T* phase or *T*-like phase) to BFO bulklike *R*-phase with increasing thickness, due to the gradually relaxed compressive strain. Undoubtedly, this structural evolution would give rise to a symmetry discrepancy for BFO thin films in various thicknesses. The differences between SHG features of 180-nm-thick BFO film and that of other three thinner films make it clear that the symmetry of epitaxial BFO thin film changes as film thickness increases.

Comparing with the characteristic SHG features of SNTO substrates, seen in Fig. 5a and g, both *p*-out and *s*-out features of 4-nm-thick BFO films are almost the same as those of SNTO substrates, while feature of *p*-out pattern for 12-nm-thick films has a little change in the intensity ratio of two bivalve structures. This similarity of SHG features for thinner BFO films and those for SNTO substrates strongly suggests that the symmetry for thinner BFO films is identical to that of SNTO surface, i.e., with *4* *mm* symmetry and the theoretical simulations also prove this. While for 100 and 180-nm-thick BFO films, both experimental and theoretical SHG results demonstrate that the symmetries of them are not *4* *mm* anymore, but in the point group *m*. We believe that the disappearance of fourfold rotational symmetry in SHG *s*-out pattern implies for the appearance of *R*-phase.

The nonlinear second-order susceptibility tensor reflects the relation between applied optical field and induced second-order polarization^34^. The tensor element *χ*_*in*_ is the contracted notation of *χ*_*ijk*_^35^, which depicts the property of polarization in direction 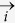 generated by field in direction 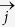 and 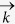, in that second-order process. It can be easily figured out that once *χ*_*31*_ and *χ*_*15*_ are equal, the material would have a higher symmetry. Therefore, when the ratio value of *χ*_*31*_/*χ*_*15*_ is approaching to 1, the structure is tending to have a higher symmetry, at least in *x* and *z* directions. This tendency is also valid on *χ*_*31*_/*χ*_*11*_ for *R-*like phase of BFO films. The calculated experimental results shown in Table 1 verify this deduction and demonstrate that the similarity of BFO thin films to *T-*like phase structure decreases with an increase of thickness.

## Summary

In summary, the crystal structure symmetry of a series of the BiFeO_3_ thin films have been investigated by XRD and SHG techniques. The XRD RSM results suggest that the structure of BFO films with the thickness less than 60 nm is with a *T*-like phase. The evolution of SHG features of BFO thin films with thicknesses of 4, 12, 60, 100, and 180 nm indicates that their structural symmetry gradually changes from *T*-like to *R*-like as the thickness increases and the appearance of *R-*phase is always accompanied by the disappearance of fourfold rotational symmetry in SHG *s*-out pattern of the sample. The nonlinear coefficient ratios for *T*-like and *R*-like BFO films are calculated, and approach to 1 of the ratio *χ*_*31*_*/χ*_*15*_ for thinner films indicates an increase of symmetry with the decrease of the thickness for BFO thin films. Our experimental results also prove that SHG can be a convenient, noninvasive, and effective method in investigating phase-related symmetry of the ferroelectric perovskite oxide films.

## Methods

### Sample fabrication

The BFO thin films with thicknesses of 4, 12, 60, 100, and 180 nm were epitaxially grown on (001)-oriented SNTO substrates under an oxygen pressure of 2 Pa at 560 °C by a laser molecular-beam epitaxy (LMBE) system, using an excimer XeCl laser with a wavelength of 308 nm, energy density of ~1.5 J/cm^2^, and repetition rate of 2 Hz. After the process of deposition, all samples were *in situ* annealed under the growth conditions for 30 min.

### Sample characterization

The ferroelectric nature of BFO films on SNTO substrates was characterized by piezoresponse force microscopy (PFM) performed on a commercial atomic force microscope (AFM, Asylum Research MFP-3D). The PFM images were collected and recorded using a Ti/Ir-coated Si cantilever (Olympus Electrilever) with a nominal ~2 N/m spring constant and a free air resonance frequency of ~75 kHz. The X-ray diffraction (XRD) and the high-solution XRD reciprocal space mapping (RSM) techniques were employed to analyze the structures of these BFO films, using the Rigaku SmartLab (9 kW) High-resolution (Ge 220 × 2) X-Ray Diffractometer with 1.54 Å X-rays.

### Second-Harmonic Generation (SHG) measurement

We carried out a systematic SHG study with a typical reflected SHG setup shown in Fig. 4. The incident laser is generated from a Ti:Sapphire oscillator with a central wavelength at 800 nm, pulse duration of 120 fs, and repetition of 82 MHz. The energy of incident light was attenuated to 70 mW before being focused. We used two configurations based on the different polarization combinations of incident and reflected light. *P*-out is denoted for the analyzer polarization parallel to the plane of incidence and incident light polarization being rotated, and *s*-out as the analyzer polarization perpendicular to the plane of incidence and incident light polarization being rotated. Single photon counting technique is conducted to count second-harmonic photons, indicating the intensity of second-harmonic signal generated from samples.

### SHG analysis

As the simplest example of nonlinear optic process, optical SHG response is a frequency-doubled light wave emitted by the polarization *P* in material induced by fundamental incident light wave, whose intensity can usually be written as *I*_*(2ω)*_ ∝ *|P*_*(2ω)*_*|*^2^. Considering the electric-dipole approximation, this process only occurs at the regions where the inversion symmetry is broken.

Under the coordinate shown in Fig. 4, the SHG intensity *I*_*(2ω)*_ reflected from BFO thin films and the corresponding sum of photons *S*_*(2ω)*_ are^34^:


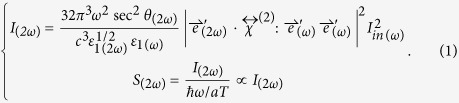


Here 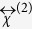 is the nonlinear susceptibility whose independent nonvanishing elements can be acquired from the previous reports^23^,^34^. *T* and *a* denote for the pulse width and the cross-sectional area of laser beam, respectively. *ε*_*1*_ and *c* are the permittivity and the speed of light in air, respectively. 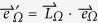, where 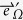 denotes for the unit polarization vector of electric field with light frequency *Ω*, and 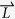 is the transmission Fresnel factor, which has diagonal elements


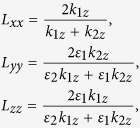


where *ε*_*2*_ is the permittivity of BFO film. *k*_*1z*_ denotes for the projection of the wave vector along *z*-axis in air, and *k*_*2z*_ is that in BFO film.

The symmetry of substrate STO (001) surface is *4* *mm*^26^, so is the slightly Nb-doped STO substrate. As we mentioned above, with the epitaxial BFO film being very thin, the symmetry of the film is approximately as the same as that of substrate. For symmetry *4* *mm*, the second-order susceptibility tensor has a form of^34^


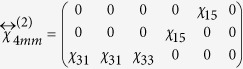


Substituting it into the Equations (1), the relation between SHG intensity and polarization angle of incident light, we can eventually obtain following two expressions


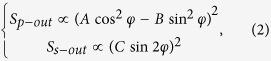


where


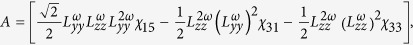



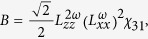



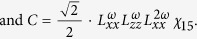


Here *φ* stands for the polarization angle of incident fundamental light.

The structure of *R*-like BFO belongs to the point group *m* whose second-order susceptibility tensor has a form of^34^


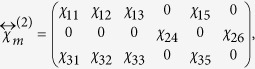


when the mirror plane of the film is aligned parallel to the incident plane. Then we can obtain corresponding SHG intensity for monoclinic BFO in the coordinate defined by Fig. 4,


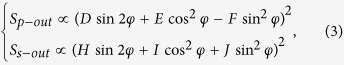


where


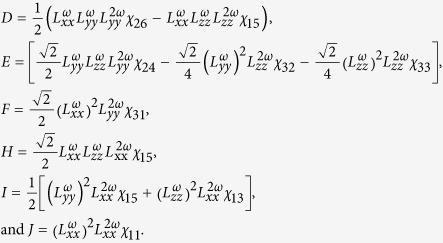


The equation for the ratio of optical nonlinear tensor *χ*_*31*_/*χ*_*15*_ in *4* *mm* symmetry is


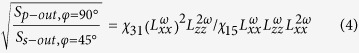


where 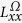, 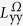 and 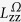 are three nonvanishing diagonal elements of transmission Fresnel tensor at frequency *Ω*^34^. Based on the definition of 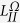, *ε*_*1*_ ≈ *1*, and *ε* ≈ *n*^*2*^, equation (4) can be written as


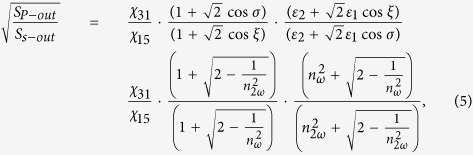


where *n*_*ω*_ and *n*_*2ω*_ are refractive indexes of BFO film for the photon energy of 1.55 eV (800 nm) and 3.11 eV (400 nm), respectively. *ξ* and *σ* are the refraction angles for 800 nm light and 400 nm light, respectively, roughly assumed as constants for various thickness in this paper. Substituting *n*_*ω*_ ≈ 2.75 and *n*_*2ω*_ ≈ 3.25^29^ into equation (5), we can obtain the evolution of *χ*_*31*_*/χ*_*15*_ as increasing the thickness of BFO films, as shown in Table 1. The nonlinear coefficient ratio *χ*_*31*_/*χ*_*11*_ for 180-nm-thick BFO film was acquired by extracting SHG intensity at *φ* = 90° both for its *p*-out and *s*-out features, also shown in Table 1.

## Additional Information

**How to cite this article**: Wang, J.-s. *et al*. Evolution of structural distortion in BiFeO_3_ thin films probed by second-harmonic generation. *Sci. Rep.* **6**, 38268; doi: 10.1038/srep38268 (2016).

**Publisher’s note:** Springer Nature remains neutral with regard to jurisdictional claims in published maps and institutional affiliations.

## Figures and Tables

**Table 1:** Nonlinear optical tensor ratio *χ*_*31*_/*χ*_*15*_ of the three thinner BFO films.

Symmetry	Thickness (nm)	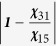
*4* *mm*	4	0.67 ± 0.03
	12	0.84 ± 0.06
	60	0.87 ± 0.09
		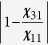
*m*	180	1.75 ± 0.09

The nonlinear optical tensor ratio *χ*_*31*_/*χ*_*11*_ for 180-nm-thick BFO film is also listed.
